# Evaluation of pentacyclic triterpenes found in *Perilla frutescens* for inhibition of skin tumor promotion by 12-*O*-tetradecanoylphorbol-13-acetate

**DOI:** 10.18632/oncotarget.5751

**Published:** 2015-10-15

**Authors:** Jiyoon Cho, Lisa Tremmel, Okkyung Rho, Andrew M. Camelio, Dionicio Siegel, Thomas J. Slaga, John DiGiovanni

**Affiliations:** ^1^ Division of Pharmacology and Toxicology in College of Pharmacy, The University of Texas at Austin, Austin, TX, USA; ^2^ Department of Nutritional Sciences, The University of Texas at Austin, Austin, TX, USA; ^3^ Department of Chemistry, The University of Texas at Austin, Austin, TX, USA; ^4^ Skaggs School of Pharmacy and Pharmaceutical Sciences, University of California San Diego, La Jolla, CA, USA; ^5^ Department of Pharmacology, The University of Texas Health Science Center at San Antonio, San Antonio, TX, USA

**Keywords:** pentacyclic triterpenes, ursolic acid, P. frutescens, chemoprevention, skin tumor promotion

## Abstract

A series of pentacyclic tritperpenes found in *Perilla frutescens* (*P. frutescens*), including ursolic acid (UA), oleanolic acid (OA), corosolic acid (CA), 3-epi-corosolic acid (3-epiCA), maslinic acid (MA), and 3-epi-maslinic acid (3-epiMA) were evaluated for their effects on epidermal cell signaling, proliferation, and skin inflammation in relation to their ability to inhibit skin tumor promotion by 12-*O*-tetradecanoylphorbol-13-acetate (TPA) and compared to UA as the prototype compound. All compounds were given topically 30 min prior to each TPA application and significantly inhibited skin tumor promotion. 3-epiCA and MA were significantly more effective than UA at inhibiting tumor development. All of these compounds significantly inhibited epidermal proliferation induced by TPA, however, CA, 3-epiCA and MA were more effective than UA. All compounds also reduced skin inflammation (assessed by infiltration of mast cells and T-cells) and inflammatory gene expression induced by TPA, however, 3-epiCA and MA were again more effective than UA. The greater ability of 3-epiCA and MA to inhibit skin tumor promotion was associated with greater reduction of Cox-2 and Twist1 proteins and inhibition of activation (i.e., phosphorylation) of IGF-1R, STAT3 and Src. Further study of these compounds, especially 3-epiCA and MA, for chemopreventive activity in other cancer model systems is warranted.

## INTRODUCTION

According to the American Cancer Society, there will be an estimated 1,658,370 new cancer cases diagnosed and 588,430 cancer deaths in the US in 2015. In addition, cancer remains the second most common cause of death in the US, accounting for nearly 1 of every 4 deaths [1]. In the mid-1970s, Michael Sporn created the term ‘chemoprevention’, which is defined as the use of natural or synthetic agents to reverse, inhibit or slow the process of carcinogenesis [2]. Chemoprevention may involve interruption of the multi-stage carcinogenesis process during tumor initiation, promotion, and/or progression [3]. Various tumor models have been used to evaluate cancer preventive agents. The multi-stage skin carcinogenesis model is a well-established model of epithelial carcinogenesis with distinct and definable stages of tumor development [4, 5]. This model can be used to evaluate cancer chemopreventive agents on each individual stage of the carcinogenesis process and is particularly useful for identifying potential mechanisms of chemopreventive action.

*Perilla frutescens* (*P. frutescens*) belongs to the annual mint family and is an edible plant frequently used in Asian countries including Korea, Japan and China. It has a pleasant flavor and taste and is used as a food ingredient. For example, perilla leaves can be added to fish, rice, soup, and vegetables and can also be pickled for many dishes. The perilla leaf extract contains a number of constituents that have pharmacologic activity, such as triterpenoids, rosmarinic acid, luteolin, caffeic acid, apigenin, and beta-carotene [6–8]. These compounds have various biological activities reported such as anti-oxidant, hepatoprotective, anti-obesity, and anti-allergic activity [7, 9–13]. Recently, emerging evidence has shown perilla extract has anti-inflammatory [7, 8, 14] and anti-cancer effects [6, 13, 15, 16].

Ursolic acid (UA) is a natural pentacyclic triterpene compound present in various edible plants including *P. fructescens* [17–21]. It has potent cancer chemopreventive activity and possesses a wide range of pharmacological activities. UA is widely studied for its apoptotic, anti-inflammatory, and anti-tumorigenic properties, including the ability to inhibit skin tumor promotion in the mouse skin model [8, 21–24]. A number of mechanisms have been attributed to the ability of UA to inhibit tumor development in these various model systems. UA has been shown to suppress multiple cell signaling pathways including, growth factor receptor activation (e.g., EGFR), and signaling through IKK/NF-kB, Akt/mTOR, Cox-2, STAT3, MMP9, and VEGF [20, 21, 25]. In addition, UA has been shown to alter levels of Bax and caspases and increase the activation of tumor suppressor proteins such as p53 and AMPK [20, 26].

In addition to UA, a number of other pentacylic triterpenes including oleanolic acid (OA), corosolic acid (CA), 3-epi-corosolic acid (3-epiCA), maslinic acid (MA), 3-epi-maslinic acid (3-epiMA), tormentic acid (TA), pomolic acid (PA), hyptadienic acid (HA), and augustic acid (AA) are found in *P. frutescens* [8]. These compounds are triterpenoid carboxylic acids with molecular formula C_30_H_48_O_x_ having six isoprene units and are synthesized in *P. frutescens* by cyclization of squalene. Banno *et al*. have reported that all of these compounds exhibited anti-inflammatory effects against TPA-induced ear edema [8]. They also have shown that UA, CA, 3-epiCA, TA, and 3-epiMA have potent inhibitory effects on TPA-induced Epstein-Barr virus early antigen activation [8]. Of the compounds found in *P. frutescens*, only TA, OA and UA, have previously been evaluated for inhibition of skin tumor promotion by TPA [8, 22–24].

In the current study, we examined the effect of 7 different triterpenes including UA, OA, AA, CA, 3-epiCA, MA, and 3-epiMA present in *P. frutescens* on epidermal proliferation, skin inflammation, inflammatory gene expression and epidermal signaling pathways induced by TPA. Six of these compounds were also evaluated for their ability to inhibit skin tumor promotion by TPA. Several of the compounds, especially 3-epiCA and MA, were found to be more effective for inhibition of skin tumor promotion than UA and are considered excellent candidates for further study of their chemopreventive effectiveness in other cancer models.

## RESULTS

### Effect of pentacyclic triterpenes found in *P. frutescens* on skin tumor promotion by TPA

**Figure 1:**
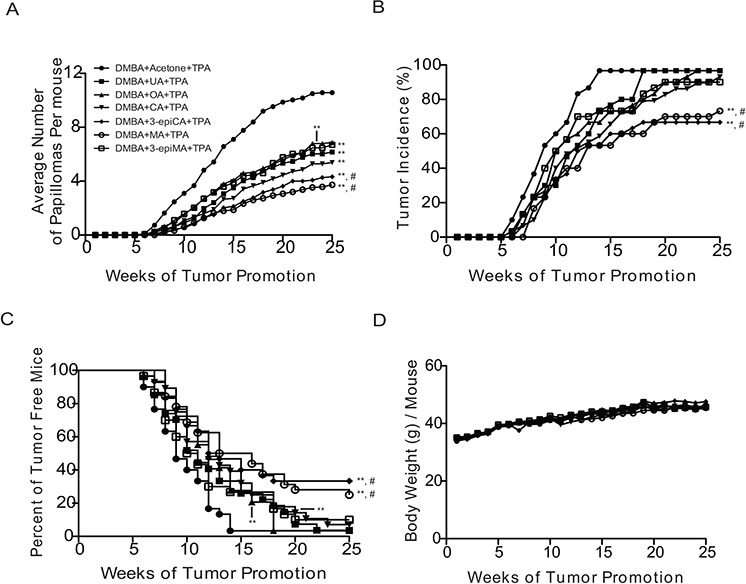
Anti-skin tumor promoting effects of UA and related triterpenes found in *P. frutescens* Female ICR mice 7 weeks old (*n* = 30/group) were initiated with 25 nmol DMBA. Two weeks after initiation with DMBA, mice were pretreated with either acetone vehicle (0.2 ml), UA (2 μmol), OA (2 μmol), CA (2 μmol), 3-epiCA (2 μmol), MA (2 μmol) and 3-epiMA (2 μmol) 30 min prior to each 6.8 nmol TPA treatment. All treatments were given twice-weekly. The number and incidence of papillomas as well as average body weights were measured once weekly for 25 weeks. **A.** Effect of UA and related triterpenes found in *P. frutescens* on tumor multiplicity (i.e., Y-axis on the graph shows the average number of papillomas per mouse). **B.** Effect of UA and related triterpenes found in *P. frutescens* on tumor incidence. **C.** Effect of UA and related triterpenes found in *P. frutescens* on tumor latency. **D.** Average body weight (g) per mouse. No significant difference was observed in body weight between triterpenes-untreated group and triterpenes-treated groups. **p ≤ 0.05 when compared to TPA-treated group; and #*p* ≤ 0.05 when compared to UA+TPA-treated group. Mann-Whitney *U* test was used for tumor multiplicity and body weight. For tumor incidence, Fisher's exact test was used. Statistical analysis of tumor latency (i.e., tumor free survival) was performed using the Mantel-Cox test.

To evaluate the anti-tumor promoting effect of UA and related triterpenes (i.e., OA, CA, 3-epiCA, MA, and 3-epiMA) present in *P. frutescens*, a two-stage skin carcinogenesis experiment was conducted using female ICR mice. AA was not included in the tumor experiment due to insufficient amount of this compound for evaluation. The control group in this experiment that was initiated with 7, 12-dimethylbenz[a]anthracene (DMBA) and promoted with TPA had 10.57 papillomas per mouse (see Figure 1A, Supplemental Figure 1, and Table 1). All of the pentacylic triterpenes evaluated in this experiment significantly inhibited skin tumor promotion by TPA. The topical dose of 2 μmol for each of the triterpenes was chosen for these experiments based on our recent study using a similar dose of UA for inhibition of skin tumor promotion [23]. Pretreatment with UA resulted in a 42% inhibition of papilloma formation (6.17 papillomas per mouse; *p* < 0.05, Mann-Whitney *U* test) (Table 1). OA (35% inhibition; 6.87 papillomas per mouse) and 3-epiMA (37% inhibition; 6.7 papillomas per mouse) also significantly inhibited skin tumor promotion by TPA (*p* < 0.05; Mann-Whitney *U* test), however, these two compounds were not more effective than UA (*p* > 0.05, Mann-Whitney *U* test). Of the remaining compounds evaluated, CA significantly inhibited TPA promotion by 49% (5.38 papillomas per mouse; *p* < 0.05, Mann-Whitney *U* test) when compared to TPA-only group but this was not significantly different compared to the group pretreated with UA. On the other hand, both 3-epiCA and MA inhibited skin tumor promotion by TPA to a greater extent than UA. In this regard, mice pretreated with 3-epiCA and MA exhibited 4.33 and 3.73 papillomas per mouse, respectively giving a 59% and 65% inhibition in tumor multiplicity (*p* < 0.05; Mann-Whitney *U* test compared to the TPA only group and the UA + TPA group, respectively) (see again Figure 1A and Table 1).

**Table 1:** Effect of UA and related triterpenes present in *P. frutescens* on tumor multiplicity and tumor incidence

Experimental groups	Average number of papillomas per mouse ± SEM	% Inhibition	% Tumor incidence
**DMBA + Acetone + TPA**	10.57 ± 1.1	-	97
**DMBA + UA + TPA**	6.17 ± 0.46 ^**^	42	97
**DMBA + OA + TPA**	6.87 ± 0.62 ^**^	35	97
**DMBA + CA + TPA**	5.38 ± 1.1 ^**^	49	93
**DMBA + 3-epiCA + TPA**	4.33 ± 0.8 ^**^, ^#^	59	67 ^**^, ^#^
**DMBA + MA + TPA**	3.73 ± 0.6 ^**^, ^#^	65	73 ^**^, ^#^
**DMBA + 3-epiMA + TPA**	6.7 ± 1.1 ^**^	37	90

** *p* ≤ 0.05 when compared to TPA-treated group; and

# *p* ≤ 0.05 when compared to UA + TPA-treated group.

Mann-Whitney *U* test and Fischer's exact test were used for tumor multiplicity and tumor incidence, respectively

As shown in Figure 1B, the incidence of papillomas in the group treated with TPA only was 97% at week 25 (Figure 1B and Table 1). Pretreatment with UA, OA, CA and 3-epiMA did not significantly reduce the overall incidence of papillomas compared to the TPA-only treated group (*p* > 0.05, Fisher's exact test). However, pretreatment with 3-epiCA and MA significantly reduced the overall tumor incidence (67% and 73%, respectively) and the reduction was statistically significant (*p* < 0.05, Fisher's exact test) when compared to TPA only group or the UA-pretreated group (both 97% incidence).

As shown in Figure 1C, tumor latency was also significantly affected by pretreatment with the various triterpenes. In this regard, pretreatment with UA, OA, CA, 3-epiCA, 3epiMA and MA significantly delayed tumor development when compared to TPA-only treated group (*p* < 0.05; Mantel-Cox test). Notably, a greater increase in tumor latency was observed in the groups of mice pretreated with 3-epiCA or MA compared to the UA-pretreated group (*p* < 0.05; Mantel-Cox test).

In summary, all of the triterpene compounds tested in this experiment significantly inhibited skin tumor promotion by TPA. UA, along with OA, CA, 3-epiCA, MA, and 3-epiMA, effectively inhibited the formation of papillomas promoted by TPA. Notably, 3-epiCA and MA exhibited the greatest inhibitory effect on papilloma multiplicity, incidence, and latency and these two compounds were significantly more effective at inhibiting skin tumor promotion when compared to the UA-pretreated group. As shown in Figure 1D, there were no significant differences in body weight between any of the triterpene-treated groups and the TPA-only treated group (*p* > 0.05, Mann-Whitney *U* test). In addition, there were no signs of epidermal toxicity in mice treated with the triterpenes indicating that the topical dosage of triterpenes used in this experiment was safe and well-tolerated.

### Effect of UA and related triterpenes found in *P. frutescens* on TPA-induced epidermal hyperproliferation

**Figure 2:**
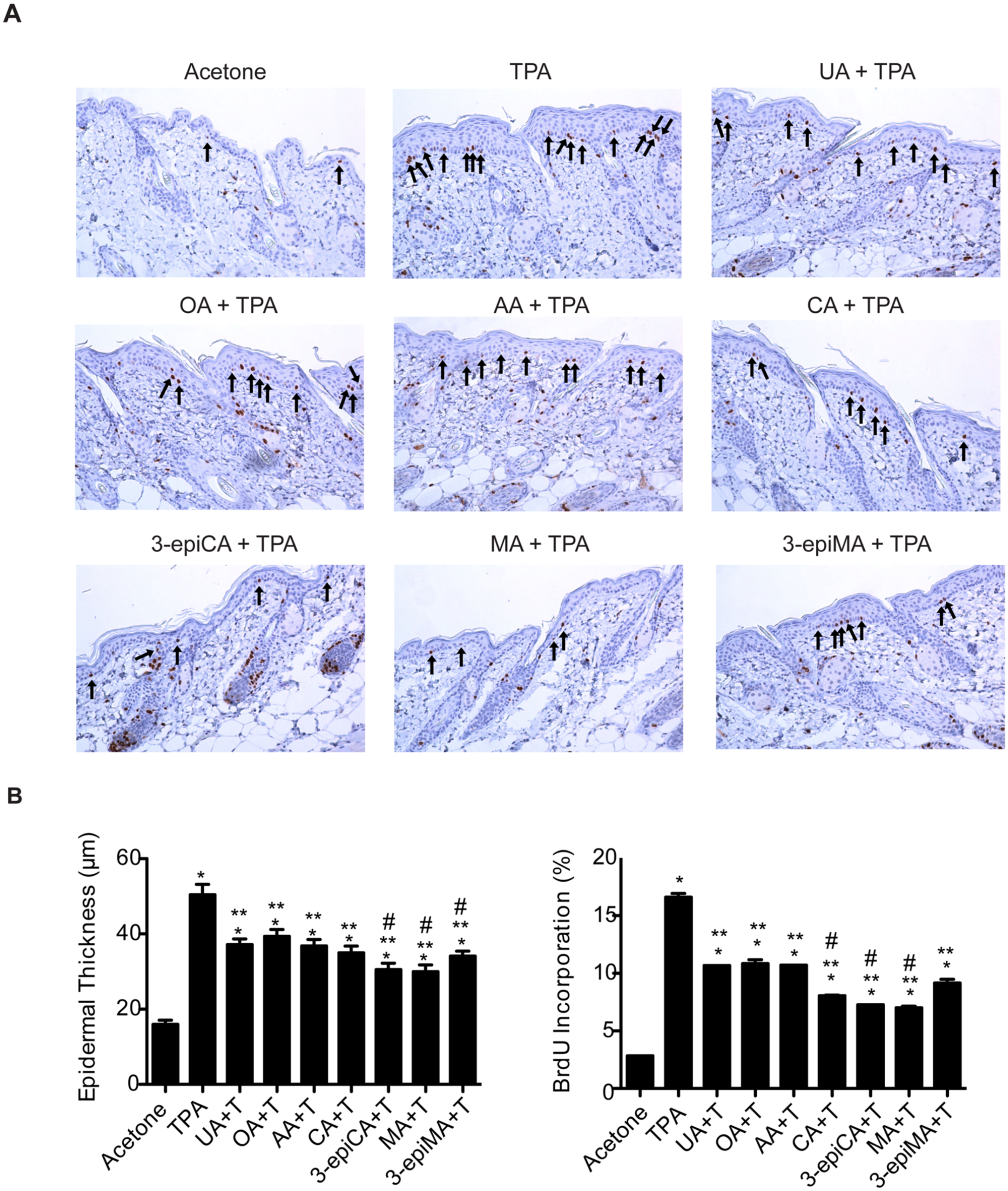
Effects of UA and related triterpenes from *P. frutescens* on TPA-induced epidermal hyperproliferation in female ICR mice The dorsal skin of mice (7–9 weeks of age; *n* = 4/group) was shaved and then two days later treated with either acetone vehicle (0.2 ml), UA (2 μmol), OA (2 μmol), AA (2 umol), CA (2 μmol), 3-epiCA (2 μmol), MA (2 μmol) or 3-epiMA (2 μmol) 30 min prior to 6.8 nmol TPA. All treatments were given twice-weekly for two weeks. Forty eight hours after the last TPA treatment, dorsal skin was fixed in 10% formalin-buffered solution, embedded in parafin and sectioned (4 μm) for BrdU-staining. **A.** Representative BrdU-stained skin sections (20X magnification). Arrows indicate BrdU-positive cells. **B.** Quantitative analyses of the effect of UA and related triterpenes found in *P. frutescens* on TPA-induced epidermal thickness and labeling index (% of BrdU-positive cells). The graphs represent mean ± standard error of the mean (SEM). *p ≤ 0.05 when compared to acetone-treated group; **p ≤ 0.05 when compared to TPA-treated group; and #*p* ≤ 0.05 when compared to UA+TPA-treated group. Mann-Whitney *U* test was used for statistical analysis.

Since epidermal proliferation is important for skin tumor development during tumor promotion in the two-stage skin carcinogenesis model [4, 5], histologic analyses were conducted to determine the effects of UA and the other triterpenes on TPA-induced epidermal hyperplasia (epidermal thickness) and labeling index (LI). The short-term treatment protocol was used for these experiments. As shown in Figure 2A and 2B, TPA treatment increased both epidermal thickness and LI 48 h after the last treatment when compared to vehicle (acetone)-treated group (*p* < 0.05; Mann-Whitney *U* test). Pretreatment with all of the triterpenes reduced both epidermal thickness and LI compared to the TPA-only treated group (*p* < 0.05, Mann-Whitney *U* test). However, 3-epiCA, MA and 3-epiMA inhibited epidermal thickness to a greater extent than UA and CA, 3-epiCA, and MA also reduced the LI to a greater extent than UA (*p* < 0.05; Mann-Whitney *U* test). Overall, these data indicate that all compounds examined in this experiment effectively inhibited TPA-induced epidermal hyperproliferation with several compounds, especially 3-epiCA and MA more effective than UA.

### Effect of triterpenes on skin inflammation induced by TPA

**Figure 3:**
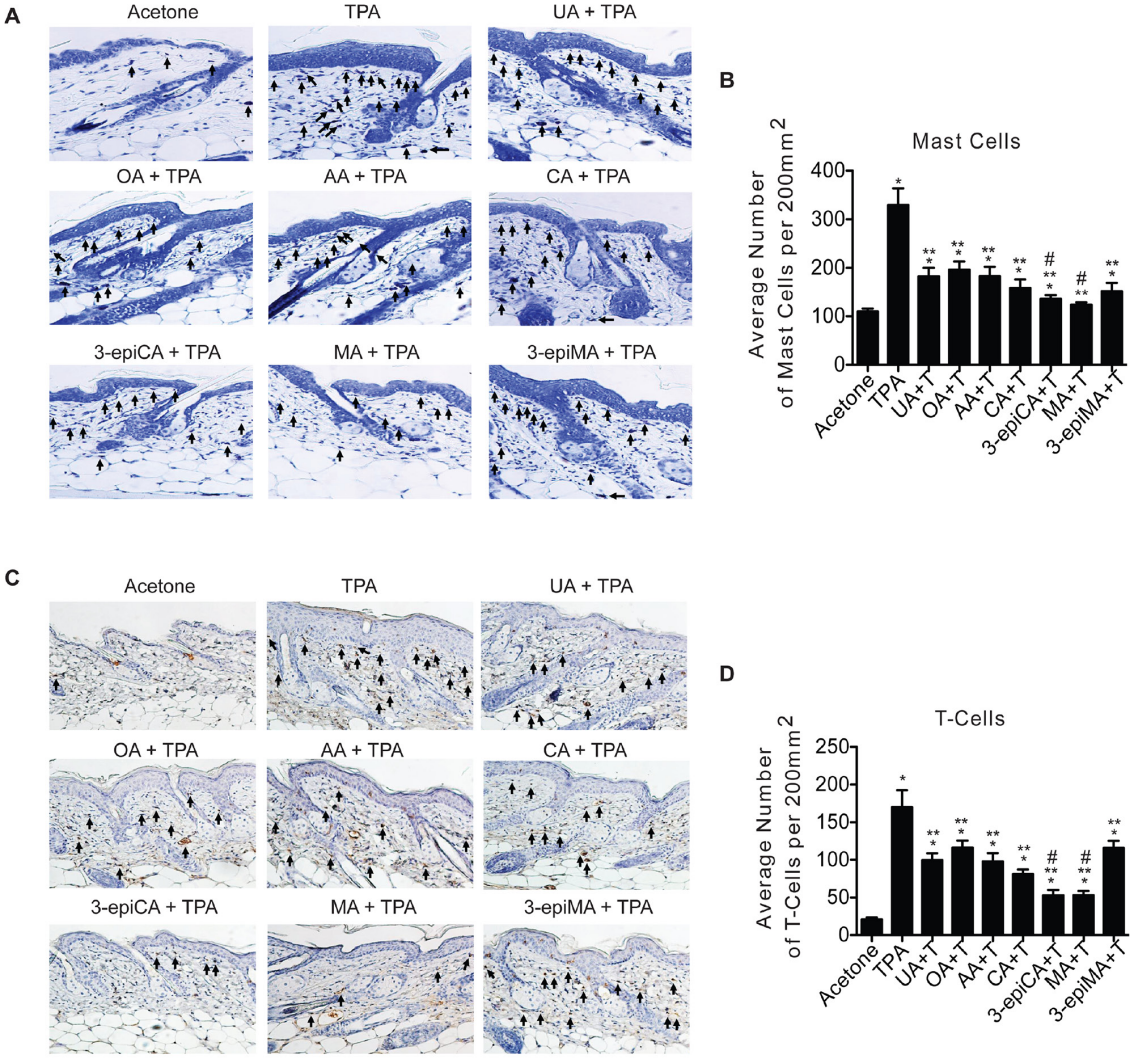
Effects of UA and related triterpenes found in *P. frutescens* on skin inflammation induced by TPA Groups of female ICR mice (7–9 weeks of age; *n* = 4) were shaved on the dorsal skin and two days later were treated with acetone vehicle (0.2 ml), UA (2 μmol), OA (2 μmol), AA (2 umol), CA (2 μmol), 3-epiCA (2 μmol), MA (2 μmol) or 3-epiMA (2 μmol) 30 min before 6.8 nmol TPA treatment. All treatments were given twice-weekly for two weeks. Dorsal skin was collected 48 hrs after the last TPA treatment, fixed in formalin, embedded in paraffin, and 4 μm sections stained with toluidine blue O solution or CD3 antibody. **A.** Representative toluidine blue O stained skin sections (20X magnification). Arrows indicate mast cells. **B.** Quantitative evaluation of the effect of UA and other triterpenes found in *P. frutescens* on TPA-induced mast cells infiltration in the dermis. The graphs represent mean ± SEM. **C.** Representative histologic skin sections of CD3^+^ staining (20X magnification). Arrows indicate T-cells. **D.** Quantitative evaluation of the effect of UA and other triterpenes found in *P. frutescens* on TPA-induced T-lymphocyte infiltration in dermis. *p ≤ 0.05 when compared to acetone-treated group; **p ≤ 0.05 when compared to TPA-treated group; and #*p* ≤ 0.05 when compared to UA + TPA-treated group. Mann-Whitney *U* test was used for statistical analysis.

The ability of UA, OA, AA, CA, 3-epiCA, MA and 3-epiMA to inhibit TPA-induced skin inflammation was first evaluated by examining effects on the dermal infiltration of inflammatory cells (i.e., mast cells and CD3 positive T-lymphocytes). As shown in Figure 3A and 3B, all compounds significantly reduced the number of mast cells (toluidine blue O-stained cells) in the dermis in the range from 40–62% (*p* < 0.05; Mann-Whitney *U* test) compared to the TPA only treated group. Again, 3-epiCA and MA were the most effective at reducing the number of infiltrated mast cells and showed a significantly greater inhibitory effect compared to the UA-pretreated group (*p* < 0.05, Mann-Whitney *U* test). Similar results were also observed for the number of infiltrated T-lymphocytes (CD3 positive cells) induced by TPA where all of the triterpenes significantly reduced their number compared to the TPA-only group (*p* < 0.05, Mann-Whitney *U* test) (Figure 3C and 3D). Again, 3-epiCA and MA were the most effective compounds at inhibiting dermal T-cell infiltration induced by treatment with TPA when compared to UA (*p* < 0.05; Mann-Whitney *U* test).

### Effect of triterpenes on TPA-induced inflammatory gene expression

**Figure 4:**
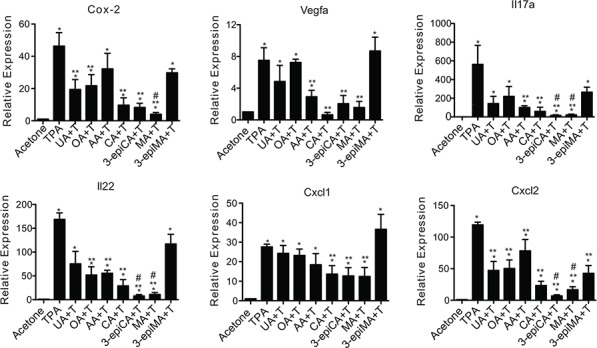
Effects of UA and a series of related triterpenes found in *P. frutescens* on TPA-induced inflammatory gene expression Female ICR mice (7–9 weeks of age; *n* = 4/group) were shaved on the dorsal skin and the two days later pretreated with acetone vehicle (0.2 ml), UA (2 μmol), OA (2 μmol), AA (2 umol), CA (2 μmol), 3-epiCA (2 μmol), MA (2 μmol) or 3-epiMA (2 μmol) before TPA treatment. All treatments were given twice-weekly for two weeks. Mice were sacrificed 6 hrs after the last TPA treatment, and epidermal RNA was isolated to be subjected to qRT-PCR analysis. mRNA levels of Cox-2, Il17a, Il22, Cxcl1, Cxcl2, and Vegfa were normalized to Gapdh. The graphs represent mean ± SEM. *p ≤ 0.05 when compared to acetone-treated group; **p ≤ 0.05 when compared to TPA-treated group; and #*p* ≤ 0.05 when compared to UA + TPA-treated group. Mann-Whitney *U* test was used for statistical analysis.

As shown in Figure 4, qRT-PCR analyses showed that UA and the other related triterpenes inhibited TPA-induced inflammatory gene expression in the epidermis. In this regard, mRNA levels of the following genes were evaluated: Cox-2, Il17a, Il22, Cxcl1, Cxcl2, and Vegfa. The increased expression of Cox-2 mRNA following TPA treatment was significantly reduced by pretreatment with UA, OA, CA, 3-epiCA and MA (*p* < 0.05; Mann-Whitney *U* test) (Figure 4). MA pretreatment led to the greatest reduction in Cox-2 mRNA and this reduction was greater than that observed in the UA pretreated group (*p* < 0.05; Mann-Whitney *U* test). Furthermore, the induction of Vegfa by TPA was lowered significantly by pretreatment with AA, CA, 3-epiCA, and MA (*p* < 0.05; Mann-Whitney *U* test). mRNA levels of Il17a and Il22 were also evaluated in this experiment. TPA treatment significantly increased the expression of both Il17a and Il22 (*p* < 0.05; Mann-Whitney *U* test) (Figure 4). Notably, the increased expression of Il17a was significantly reduced in the groups pretreated with AA, CA, 3-epiCA, and MA, and a greater inhibitory effect was observed in the 3-epiCA- and MA-pretreated groups (*p* < 0.05; Mann-Whitney *U* test) compared to the UA treated group. In addition, OA, AA, CA, 3-epiCA, and MA significantly inhibited TPA-induced Il22 expression (*p* < 0.05; Mann-Whitney *U* test), while UA and 3-epiMA did not show a statistically significant decrease in the expression of Il22 induced by TPA. Again, 3-epiCA and MA produced the greatest inhibition of Il22 expression.

We also investigated the effects of the triterpenes on proinflammatory chemokines, Cxcl1 and Cxcl2. As shown in Figure 4, the expression Cxcl1 and Cxcl2 was significantly increased by TPA treatment (*p* < 0.05; Mann-Whitney *U* test). Pretreatment with CA, 3-epiCA, and MA inhibited Cxcl1 mRNA induction. In addition, all of the compounds significantly inhibited the expression of Cxcl2 by TPA (*p* < 0.05; Mann-Whitney *U* test). Notably, 3-epiCA and MA, were most effective and produced greater inhibition than UA on Cxcl2 mRNA expression (*p* < 0.05, Mann-Whitney *U* test).

Collectively, these data demonstrate that UA and related triterpenes found in *P. frutescens* inhibited inflammatory gene expression in the epidermis, although some of these genes were differentially affected by the individual triterpenes. Notably, when looking at all the genes examined, 3-epiCA and MA produced the most significant effects on the largest number of inflammatory genes induced by TPA.

### Effect of triterepenes on epidermal signaling pathways induced by TPA

**Figure 5:**
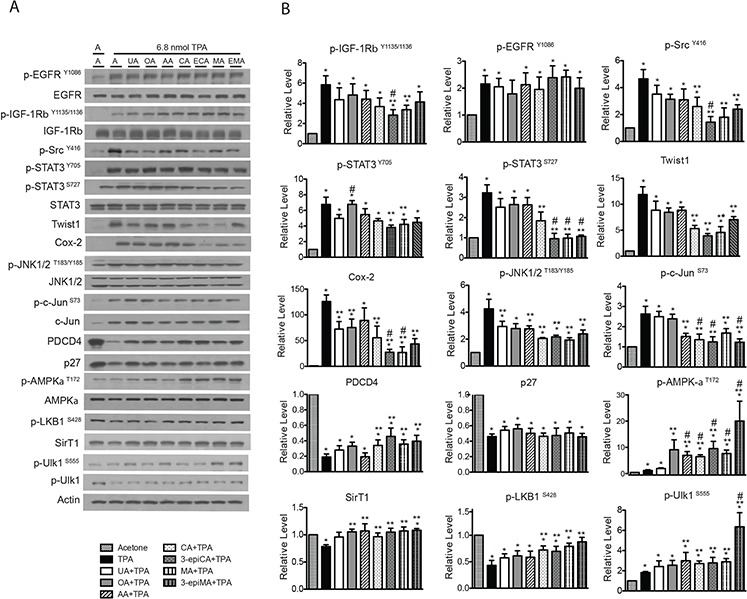
Effects of UA and related triterpenes from *P. frutescens* on TPA-induced epidermal signaling pathways in female ICR female Groups of mice (*n* = 4/group) were shaved on the dorsal skin and then two days later treated with acetone vehicle, UA (2 μmol), OA (2 μmol), AA (2 umol), CA (2 μmol), 3-epiCA (ECA, 2 μmol), MA (2 μmol) or 3-epiMA (EMA, 2 μmol) 30 min prior to 6.8 nmol TPA treatment. All treatments were given twice-weekly for two weeks. Six hrs after the last TPA treatment, epidermal lysates were prepared for Western blot analysis. **A.** Representative Western blots for the effect UA and related triterpenes on TPA-induced signaling pathways. **B.** Quantitative evaluation of the Western blot analysis. Phosho-proteins are normalized to both total protein and actin. The levels of twist1, Cox-2, Pdcd4, p27 and SirT1 were normalized to actin. Note that multiple gels were run to quantitate the signaling proteins shown. Actin blots were run and probed for each individual gel, and all proteins were normalized to the corresponding actin blot for that gel. The actin blots shown in A are presented as a representative blot. The graphs in B represent mean ± SEM of three independent experiments. *p ≤ 0.05 when compared to acetone-treated group; **p ≤ 0.05 when compared to TPA-treated group; and #*p* ≤ 0.05 when compared to UA+TPA-treated group. Mann-Whitney *U* test was used for statistical analysis.

The effects of topical pretreatment with UA, OA, AA, CA, 3-epiCA, MA and 3-epiMA on multiple signaling pathways induced by TPA in the epidermis were examined at a 6 hr time point (see Figure 5, panels A and B). As shown in Figure 5A and 5B, groups pretreated with UA, OA, AA, CA, or 3-epiMA showed a slight inhibition of phosphorylation of IGF-1βR^Y1135/1136^ (not statistically significant). However, a statistically significant inhibition was observed in the groups pretreated with 3-epiCA and MA. The inhibitory effect of 3-epiCA on activation of IGF-1βR^Y1135/1136^ by TPA treatment was also significantly greater when compared to the UA-pretreated group (*p* < 0.05; Mann-Whitney *U* test). On the other hand, none of the triterpene compounds showed inhibition of TPA-induced p-EGFR^Y1086^ levels. Evaluation of the effect of the triterpenes on p-Src^Y416^ levels was also examined. Significant inhibition of the phosphorylation of p-Src^Y416^ was observed in groups pretreated with CA, 3-epiCA, MA and 3-epiMA (*p* < 0.05; Mann-Whitney *U* test). Again, similar to the result of IGF-1βR signaling, 3-epiCA was more effective at reducing p-Src^Y416^ levels than UA following treatment with TPA (*p* < 0.05; Mann-Whitney *U* test).

The inhibitory effect of the triterpenes was also examined on STAT3 phosphorylation (both the Y705 and the S727 sites) induced by TPA (Figure 5A and 5B). As shown in the figure, CA, 3-epiCA, MA and 3-epiMA significantly decreased the phosphorylation of STAT3 at S727 (*p* < 0.05; Mann-Whitney *U* test) while rest of the compounds tested did not show a statistically significant reduction in p-STAT3^S727^ level when compared to the TPA-only treated group. Notably, 3-epiCA, MA, and 3-epiMA showed the greatest inhibitory effect (*p* < 0.05; Mann-Whitney *U* test). In addition, 3-epiCA and MA significantly inhibited the phosphorylation of p-STAT3^Y705^ (*p* < 0.05; Mann-Whitney *U* test) while the other compounds did not significantly alter STAT3 phosphorylation at this site. A downstream target of STAT3, Twist1, was also examined. Pretreatment with CA, 3-epiCA, MA, and 3-epiMA reduced the level of Twist1 induced by TPA (*p* < 0.05; Mann-Whitney *U* test).

Additional analyses were performed to examine the effect of the triterpenes on other signaling pathways such as Cox-2, JNK1/2, c-Jun, Pdcd4 and p27. As shown in Figure 5A and 5B, the level of Cox-2 protein was significantly reduced by all of the compounds except AA and the reduction by 3-epiCA and MA was greater than that observed in the UA-pretreated group (*p* < 0.05; Mann-Whitney *U* test). In addition, all triterpenes excluding OA significantly reduced the phosphorylation of JNK1/2^T183/Y185^ that was stimulated by TPA treatment (*p* < 0.05; Mann-Whitney *U* test), and AA, CA, 3-epiCA, MA and 3-epiMA had a significant inhibitory effect on TPA-induced c-Jun phosphorylation at S73. Notably, a greater reduction in p-c-Jun^S73^ was observed in the groups pretreated with CA, 3-epiCA, and 3-epiMA (*p* < 0.05; Mann-Whitney *U* test) compared with the UA-pretreated group. The effect of the pentacyclic triterpenes on tumor suppressor proteins, Pdcd4 and p27, was investigated. The decreased level of Pdcd4 after TPA treatment was partially reversed by CA, 3-epiCA, MA and 3-epiMA (*p* < 0.05; Mann-Whitney *U* test) whereas none of compounds had significant effects on p27 levels following treatment with TPA.

Several studies including those from our group have reported AMPK activators (e.g. Metformin, AICAR, compound C) inhibit skin cancer *in vitro* and *in vivo* [27–29], therefore the effect of the triterpenes on AMPK signaling following TPA treatment was also evaluated. As shown in Figure 5A and 5B, TPA treatment induced a modest activation of AMPK (assessed by phosphorylation at T172). Pretreatment with UA did not significantly increase AMPK activation while pretreatment with the other compounds tested significantly increased the phosphorylation of AMPK-α^T172^ above that observed following TPA treatment (*p* < 0.05; Mann-Whitney *U* test). The status of SirT1 and LKB1 was also evaluated. As shown in Figure 5A and 5B, activation of LKB1 (phosphorylation at S428) was enhanced by pretreatment with CA, 3-epiCA, MA and 3-epiMA (*p* < 0.05; Mann-Whitney *U* test) while all triterpene compounds except UA significantly increased the level of SirT1 (*p* < 0.05, Mann-Whitney *U* test). As expected, phosphorylation of the downstream target of AMPK, Ulk1 at S555 was also elevated by pretreatment with AA, CA, 3-epiCA, MA and 3-epiMA. Notably, the dramatic upregulation as seen in AMPK signaling after 3-epiMA pretreatment was also observed in the phosphorylation of Ulk1 at S555 after pretreatment with this compound.

In summary, the data in Figure 5 demonstrate that pretreatment with the various triterpenes that were evaluated caused alterations in a number of signaling pathways important for skin tumor promotion by TPA. Several of the compounds, including 3-epiCA and MA showed greater effects on a broad range of signaling pathways that may have contributed to their greater ability to inhibit skin tumor promotion by TPA.

## DISCUSSION

In the current study, all of the triterpenes tested significantly inhibited skin tumor promotion by TPA. 3-EpiCA and MA were the most effective compounds at inhibiting the development of papillomas and were more effective than the prototype and more widely studied UA. Mechanistic studies revealed that all of the tripterpenes tested had the ability to inhibit TPA-induced epidermal hyperproliferation and skin inflammation. Again, the overall inhibitory effects on epidermal proliferation and skin inflammation were the greatest in the groups pretreated with 3-epiCA or MA and significantly greater than seen in the UA + TPA group. Analysis of epidermal signaling pathways induced by TPA revealed that there were variable effects of the different compounds on individual signaling pathways. However, the greater ability of 3-epiCA and MA to inhibit skin tumor promotion was associated with greater reduction of Cox-2 and Twist1 proteins and inhibition of activation (i.e., phosphorylation) of IGF-1R, STAT3 and Src. Collectively, the current data demonstrate that triterpenes from *P. frutescens* inhibit skin tumor promotion by inhibiting signaling pathways associated with epidermal proliferation and inflammation.

Several of the pentacyclic triterpenes found in *P. frutescens* have been widely studied. In this regard, UA has been shown to have apoptotic, anti-inflammatory, and anti-tumorigenic effects [18, 20, 25, 30]. UA and OA are often found together in plants, including *P. frutescens*, and possess similar pharmacological properties [18, 20, 21, 30]. OA, like UA, also exhibits anti-inflammatory, gastro-protective, wound-healing, and anti-microbial properties [31, 32]. In addition, several studies have shown its anti-cancer effects in both *in vitro* and *in vivo* models [22, 33]. OA inhibits proliferation and induces apoptosis in many cancer cell lines including breast, lung, and skin [34–37]. CA also exists in abundance in the plant kingdom, including bananas and loquat as well as *P. frutescens* [8, 38]. A number of studies have shown that CA has anti-diabetic, anti-oxidant, anti-inflammatory, apoptotic, and anti-cancer activities. Its anti-cancer effects have not been as extensively studied *in vivo* although several *in vitro* studies have demonstrated growth inhibitory effects in cancer cell lines including gastric, colon, cervix, leukemia and lung [39–45]. MA, also known as crategolic acid, has been isolated from *P. frutescens* as well as other edible plants such as olive fruit, spinach, eggplant, mustard, basil and legumes [46]. Recently, the anti-proliferative and apoptotic properties of MA have been demonstrated in various cancer cell lines such as colon, liver, bladder, uterus and breast [19, 47–51].

As noted in the Introduction, of the compounds evaluated in the current study, only UA and OA have previously been shown to inhibit skin tumor promotion by TPA [17, 22–24]. In a recent study by our group, topical pretreatment with 2 μmol of UA reduced the number of TPA promoted skin papillomas by 39% [23]. In the current study, the same dose of UA given before TPA reduced papilloma formation by 42%. Thus, these two independent studies show that UA, when given topically to the same mice (female ICR) and under similar experimental conditions, produced essentially the same level of inhibition of tumor promotion. Tokuda *et al*. reported that topical application of OA inhibited skin tumor promotion by TPA in female ICR mice using considerably lower doses [22]. In this study, they also compared the anti-tumor promoting effect of OA with UA using two different treatment protocols. Both OA and UA inhibited skin tumor promotion by TPA to a similar extent when the compounds were given prior to each TPA application over a 20 week period. Surprisingly, both compounds also were shown to inhibit skin tumor development when given only once prior to the first TPA application. The mechanisms for the observed inhibition of skin tumor promotion by both OA and UA in either protocol were not reported in this earlier study. In the current study, UA and OA given at the same topical dose produced a very similar inhibition of skin tumor promotion (42% vs 35%, respectively; Table 1). The potential anti-cancer effects of CA and MA have been reported in a number of *in vitro* studies, however, only a few studies have evaluated their inhibitory effects in cancer models. Li *et al*. reported that subcutaneous administration of MA (10 and 50 mg/kg) inhibited growth of pancreatic cancer cells in a xenograft mouse model [52]. In another study, MA was given in the diet (100 mg/kg) to APC Min/+ mice and was shown to reduce colon carcinogenesis by 45% [53]. In addition, MA inhibited both the size and weight of bladder tumors in a xenograft mouse model [50]. Recently, Yoo *et al*. have reported that CA possess suppressive effects on angiogenesis and lymphangiogenesis *in vivo* using a CT-26 colon carcinoma animal model [54].

In contrast to UA and OA, no studies to date have reported on the anti-skin tumor promoting effects of CA, 3-epiCA, MA and 3-epiMA. Therefore, our results report for the first time the ability of these triterpenoids that are found together in *P. frustesens* to inhibit skin tumor promotion by TPA in the two-stage skin carcinogenesis model. As shown in Figure 1 and Table 1, all four of these compounds had the ability to inhibit skin tumor promotion by TPA when given topically 30 min prior to each TPA application. This was seen primarily in the ability to inhibit the formation of papillomas that were promoted by TPA. Significant effects were also observed on tumor latency. When compared to the activity of UA for inhibition of skin tumor promotion, two compounds (i.e., 3-epiCA and MA) exhibited greater ability to inhibit skin tumor promotion as assessed by effects on tumor multiplicity, tumor incidence, and tumor free survival (i.e., latency) (see again Figure 1 and Table 1). Although we did not examine the effect of the triterpene compounds on the development of squamous cell carcinomas (SCCs), papillomas are considered premalignant tumors [4, 5], and we have previously shown that reductions in the number of papillomas leads to decreases in the number of SCCs [4, 27, 55].

In our previous study, topical application of 2 μmol UA prior to TPA application was shown to significantly inhibit Cox-2, p-NF-κB p65^S536^, and p-Akt^T308^ signaling as well as reduce phosphorylation of p-Src^Y416^, p-STAT3^Y705^, and p-JNK1/2^T183/Y185^ [23]. In the current study, the effect of 2 μmol of UA, OA, AA, CA, 3-epiCA, MA, and 3-epiMA were compared for their effects on some of these same pathways as well as others (Figure 5). All compounds reduced Cox-2 expression and the level of p-JNK1/2^T183/Y185^, although the reduction in Cox-2 level with AA pretreatment was not statistically significant. In addition, all of the compounds reduced the levels of p-c-Src^Y416^, although the values with UA, OA and AA were not statistically significant. Some of the triterpenes, notably, 3-epiCA and MA produced a greater degree and a broader range of inhibition of signaling pathways. Further analyses revealed that these two compounds, in addition to inhibiting Cox-2, p-JNK1/2^T183/Y185^ and p-Src^Y416^, significantly reduced p-IGF-1R^Y1135/1136^, p-Stat3^S727^, Twist1, p-c-Jun^S73^ and increased Pdcd4, p-AMPK-α^T172^, Sirt1, p-LKB1^S428^ and p-ULK1^S555^. Thus, the greater inhibitory activity of these two compounds was associated with a greater ability to modulate multiple growth factor and inflammatory signaling pathways in the epidermis.

During the tumor promotion stage, repeated topical treatments of TPA produce and maintain chronic epidermal cell proliferation [5]. Initiated cells have a selective growth advantage during TPA induced epidermal hyperplasia and proliferation, and undergo clonal expansion to form pre-malignant papillomas [4, 5, 56]. Therefore, we examined the anti-proliferative effect of the triterpenes on TPA-induced epidermal hyperproliferation. Our previous data indicated that topical treatment of UA inhibited both epidermal thickness and LI induced by TPA [23]. In the current study, UA and the other related triterpenes significantly inhibited TPA-induced epidermal thickness and LI. However, 3-epiCA and MA, again showed the greatest effect on epidermal hyperproliferation, and these two compounds were more effective than the group pretreated with UA.

Another important aspect of tumor promotion is chronic inflammation. Upregulation and secretion of pro-inflammatory molecules by TPA treatment recruits inflammatory cells such as mast cells, monocytes, leukocytes, T-and B-lymphocytes, and macrophages into the dermis [57, 58]. The number of these cells is increased in the dermis adjacent to the epidermis, and they promote tumor growth by producing growth factors, cytokines, and chemokines [57, 58]. Data previously published from our group suggested that multiple applications of TPA increased the number of dermal infiltrated inflammatory cells such as mast cells, T-lymphocytes, and macrophages in mouse skin [23, 59]. Topical treatment with UA and other chemopreventive agents such as rapamycin and resveratrol, have been shown to decrease the number of these cells in the dermis following TPA treatment [23, 59]. In addition, Banno *et al*. demonstrated the anti-inflammatory effect of UA and some other triterpenes including OA, AA, CA, 3-epiCA, CA, MA and 3-epiMA on TPA-induced ear edema [8, 60]. In this study, they determined ID_50_ values of triterpenoids and the range of ID_50_ on TPA-induced inflammation in mouse ear was from 0.09 to 0.15 mg/ear, showing that no dramatic difference of ID_50_ was observed among the groups pretreated with UA, AA, CA, 3-epiCA, MA or 3-epiMA dissolved in MeOH-CHCl_3_-H_2_O vehicle. However, given the higher ID_50_ value (0.3 mg/ear) of OA, OA was less effective than the others. As shown in Figure 3, UA and related triterpenes significantly decreased the number of dermal mast cells and T-lymphocytes induced by TPA. Again, the most effective compounds were 3-epiCA and MA. In addition, these two compounds were the most effective overall at inhibiting TPA-induced inflammatory gene expression (Figure 4).

In conclusion, evaluation of a series of pentacyclic triterpenes found in *P. frutescens* showed that all of these compounds inhibited skin tumor promotion by TPA and that 3-epiCA and MA were more effective than UA. The inhibitory effects of all compounds and the greater effect of several compounds such as 3-epiCA and MA on skin tumor promotion were due to the reduction of epidermal hyperproliferation, skin inflammation, and alterations in a number of epidermal signaling pathways critical for tumor promotion. Although some of these compounds were previously shown to have anti skin tumor promoting activity (UA and OA), this is the first comprehensive comparison of a series of pentacyclic triterpenes found in *P. frutescens* for their effects on skin tumor promotion *in vivo* and included new compounds not previously evaluated (i.e., CA, 3-epiCA MA and 3-epiMA). Furthermore, we found that two of these compounds were more active than UA and have provided a potential mechanistic basis for this increased activity. Overall, these compounds and especially 3-epiCA and MA, deserve further evaluation for their cancer chemopreventive activity.

## MATERIALS AND METHODS

### Animals and diets

We purchased Female ICR (CD-1) 6–7 weeks of age from Harlan Laboratories Inc. (Houston, TX). Mice were group housed (*n* = 4~5/cage) for all experiments. For the short-term experiments, mice were maintained on a regular chow diet. Mice received a semi-purified diet containing 10 kcal% fat (D12450B, Research Diets Inc., New Brunswick, NJ) for tumor experiments. All animal experiments were performed according to both Institutional as well as NIH guidelines under an approved IACUC protocol.

### Chemicals

UA and OA were purchased from Sabinsa Company (East Windsor, NJ) and Stanford Chemicals (Irvine, CA), respectively. AA, CA, MA, 3-epiCA, and 3-epiMA were prepared as recently described by us [61]. All triterpenes used in the current experiments were > 98% pure. Chemical agents including DMBA and 5-Bromo-2′-deoxyurine (BrdU) were purchased from Sigma Chemical Co. (St. Louis, MO), and TPA was purchased from LC laboratories (Woburn, MA).

### Two-stage skin carcinogenesis assays

Female ICR mice (*n* = 30/group) 7–8 weeks of age were shaved on the dorsal skin and then initiated 48 hrs later with a single topical application of 25 nmol of DMBA in 0.2 ml acetone or 0.2 ml acetone vehicle. During tumor promotion (begun 2 weeks after initiation), mice received 2 μmol of UA, OA, CA, 3-epiCA, MA or 3-epiMA 30 min prior to each 6.8 nmol TPA application. TPA was administered twice-weekly for the duration of the experiment until the number of papillomas per mouse reached a plateau (25 weeks). Body weight, tumor incidence (percentage of mice with papillomas), and tumor multiplicity (average number of papillomas per mouse) were measured once a week for the duration of the experiment. In addition, the surface area of all detectable papillomas was measured by digital calipers at the termination of the experiment.

### Short-term treatment protocol

Female ICR female mice (7–8 weeks old) were shaved on the dorsal skin and two days later received topical treatments with acetone vehicle (200 μl) or 2 μmol of UA, OA, AA, CA, 3-epiCA, MA, or 3-epiMA 30 min before 6.8 nmol TPA treatment. All treatments were given twice weekly for two weeks. Epidermal tissue was then harvested 6 hrs after (for Western blot analysis and qRT-PCR) or 48 hrs after (for histologic evaluation) the last TPA treatment.

### Histologic analyses

For histologic evaluation of epidermal thickness, LI, and the number of dermal infiltrated inflammatory cells, groups of mice (*n* = 4 mice/group) were treated with the triterpene compounds according to the short-term protocol regimen. BrdU (100 μg/g B.W.) dissolved in PBS was injected *i.p.* to mice 30 min prior to sacrifice. Forty-eight hrs after the last TPA treatment, dorsal skin was excised and fixed in 10% neutral-buffered formalin. The fixed skin samples were embedded in paraffin, sectioned (4 μm) and stained with toluidine blue O (Fisher Scientific, Pittsburgh, PA), anti-BrdU (Abcam, Cambridge, MA), or anti-CD3 (Cell Signaling Technology, Beverly, MA). Epidermal thickness and LI were determined as described previously [62]. The number of inflammatory cells in the dermis was counted per 200 mm^2^ field as previously described [59].

### Collection of epidermal tissue and preparation of epidermal protein lysates and total RNA

For the preparation of epidermal protein lysates and total RNA, groups of mice (*n* = 4 mice per group) received pretreatment with acetone or triterpene compounds before TPA treatment according to the short-term treatment protocol. Mice were sacrificed 6 hrs after the last TPA treatment and epidermal protein lysates were collected as previously described [59]. Epidermal RNA samples were isolated using an RNreasy mini kit (Qiagen, Valencia, CA) as previously described [63, 64] and subjected for quantitative real-time RT-PCR (qRT-PCR) analysis.

### Western blot analysis

For Western blot analyses, the protein concentration of the supernatant was measured using the lowry protein assay kit (Thermo Scientific, Waltham, MA). Aliquots of supernatant containing 30 μg protein were boiled in sodium dodecylsulfate (SDS) sample loading buffer for 5 min before electrophoresis on 6–15% SDS-polyacrylamide gel and then transferred to the nitrocellulose membrane. The blots were blocked with 5% bovine serum albumin (BSA) or 5% non-fat dry milk in TBST buffer [TBS containing 0.1% Tween-20] for 1 hr at room temperature. The membranes were incubated overnight at 4°C with 1:1000 dilutions of primary antibodies. Blots were washed three times with TBST at 10 min interval followed by incubation with 1:5000 horseradish peroxidase-conjugated secondary antibodies (rabbit or mouse) for 1 hr and washed in TBST for three times. The transferred proteins were visualized with an ECL detection kit (Thermo Scientific, Waltham, MA) according to the manufacturer's instructions. Antibodies against the following proteins were used: IGF-1βR, p-IGF-1βR^Y1135/1136^, p-Src ^Y416^, p-JNK1/2^T183/Y185^, JNK1/2, p-c-Jun^S73^, c-Jun, Pdcd4, p-AMPK-α^T172^, AMPK-α, SirT1, p-LKB1^S416^, p-Ulk1^S555^, Ulk1, STAT3, p-STAT3^S727^, pSTAT3^Y705^ (Cell Signaling Technology, Beverly, MA); p-EGFR^Y1086^ (Abcam, Cambridge, MA); EGFR (Millipore); p27 (BD Biosciences, Bradford, MA); twist1 and actin (Sigma. St. Louis, MO).

### qRT-PCR analysis

qRT-PCR analyses were performed as previously described [65]. For preparation of cDNA using High Capacity cDNA Reverse Transcription Kits (Applied Biosystems, Grand Island, NY), 1 μg of RNA was mixed with 2 μl 10X RT buffer, 2 μl 10X random primers, 0.8 μl 25X dNTP mix (100 mM), 1 μl reverse transcriptase and RNase-free water in a total 20 μl volume. The mixtures were incubated at in the order of 25°C for 10 min, 37°C for 120 min, 85°C for 5 min, and 4°C for 5 min. For qRT-PCR analysis, cDNA (150 ng) was mixed with 5 μl of 2X iTag™ universal SYBR green supermix (Bio-Rad, Hercules, CA), 0.5 μl of 10 μM forward primers, 0.5 μl of 10 μM reverse primers, and RNase-free water in a total 10 μl volume. The mixtures were then subjected to qRT-PCR using a ViiA™ 7 (Applied Biosystems, Grand Island, NY) real time instrument and analysis software.

### Statistical analysis

The statistical evaluation of significant differences between groups was performed using the Mann-Whitney *U* test for the following data; protein expression, gene expression, epidermal thickness, labeling index, the number of dermal infiltrated inflammatory cells, tumor multiplicity, and body weight. A one-tailed Fisher's exact test was used for comparison of tumor incidence. The Mantel-Cox test was used for comparisons of tumor latency. Significance in all cases was set at *p* ≤ 0.05.

## SUPPLEMENTARY FIGURE



## References

[1] Siegel R, Ma J, Zou Z, Jemal A (2014). Cancer statistics, 2014. CA: a cancer journal for cliniciangs.

[2] Sporn MB, Suh N (2002). Chemoprevention: an essential approach to controlling cancer. Nature reviews Cancer.

[3] Surh YJ (2003). Cancer chemoprevention with dietary phytochemicals. Nature reviews Cancer.

[4] Abel EL, Angel JM, Kiguchi K, DiGiovanni J (2009). Multi-stage chemical carcinogenesis in mouse skin: fundamentals and applications. Nature protocols.

[5] DiGiovanni J (1992). Multistage carcinogenesis in mouse skin. Pharmacology & therapeutics.

[6] Osakabe N, Yasuda A, Natsume M, Yoshikawa T (2004). Rosmarinic acid inhibits epidermal inflammatory responses: anticarcinogenic effect of Perilla frutescens extract in the murine two-stage skin model. Carcinogenesis.

[7] Asif M (2012). Phytochemical study of polyphenols in Perilla Frutescens as an antioxidant. Avicenna journal of phytomedicine.

[8] Banno N, Akihisa T, Tokuda H, Yasukawa K, Higashihara H, Ukiya M, Watanabe K, Kimura Y, Hasegawa J, Nishino H (2004). Triterpene acids from the leaves of Perilla frutescens and their anti-inflammatory and antitumor-promoting effects. Bioscience, biotechnology, and biochemistry.

[9] Kim MJ, Kim HK (2009). Perilla leaf extract ameliorates obesity and dyslipidemia induced by high-fat diet. Phytotherapy research: PTR.

[10] Kim MK, Lee HS, Kim EJ, Won NH, Chi YM, Kim BC, Lee KW (2007). Protective effect of aqueous extract of Perilla frutescens on tert-butyl hydroperoxide-induced oxidative hepatotoxicity in rats. Food and chemical toxicology: an international journal published for the British Industrial Biological Research Association.

[11] Oh HA, Park CS, Ahn HJ, Park YS, Kim HM (2011). Effect of Perilla frutescens var. acuta Kudo and rosmarinic acid on allergic inflammatory reactions. Experimental biology and medicine.

[12] Shin TY, Kim SH, Kim SH, Kim YK, Park HJ, Chae BS, Jung HJ, Kim HM (2000). Inhibitory effect of mast cell-mediated immediate-type allergic reactions in rats by Perilla frutescens. Immunopharmacology and immunotoxicology.

[13] Ueda H, Yamazaki M (2001). Anti-inflammatory and anti-allergic actions by oral administration of a perilla leaf extract in mice. Bioscience, biotechnology, and biochemistry.

[14] Urushima H, Nishimura J, Mizushima T, Hayashi N, Maeda K, Ito T (2015). Perilla frutescens extract ameliorates DSS-induced colitis by suppressing proinflammatory cytokines and inducing anti-inflammatory cytokines. American journal of physiology Gastrointestinal and liver physiology.

[15] Ueda H, Yamazaki C, Yamazaki M (2003). Inhibitory effect of Perilla leaf extract and luteolin on mouse skin tumor promotion. Biological & pharmaceutical bulletin.

[16] Lin CS, Kuo CL, Wang JP, Cheng JS, Huang ZW, Chen CF (2007). Growth inhibitory and apoptosis inducing effect of Perilla frutescens extract on human hepatoma HepG2 cells. Journal of ethnopharmacology.

[17] Huang MT, Ho CT, Wang ZY, Ferraro T, Lou YR, Stauber K, Ma W, Georgiadis C, Laskin JD, Conney AH (1994). Inhibition of skin tumorigenesis by rosemary and its constituents carnosol and ursolic acid. Cancer research.

[18] Liu J (2005). Oleanolic acid and ursolic acid: research perspectives. Journal of ethnopharmacology.

[19] He X, Liu RH (2007). Triterpenoids isolated from apple peels have potent antiproliferative activity and may be partially responsible for apple's anticancer activity. Journal of agricultural and food chemistry.

[20] Shanmugam MK, Dai X, Kumar AP, Tan BK, Sethi G, Bishayee A (2013). Ursolic acid in cancer prevention and treatment: molecular targets, pharmacokinetics and clinical studies. Biochemical pharmacology.

[21] Zang LL, Wu BN, Lin Y, Wang J, Fu L, Tang ZY (2014). Research progress of ursolic acid's anti-tumor actions. Chinese Journal of Integrative Medicine.

[22] Tokuda H, Ohigashi H, Koshimizu K, Ito Y (1986). Inhibitory effects of ursolic and oleanolic acid on skin tumor promotion by 12-O-tetradecanoylphorbol-13-acetate. Cancer letters.

[23] Cho J, Rho O, Junco J, Carbajal S, Siegel D, Slaga TJ, DiGiovanni J (2015). Effect of Combined Treatment with Ursolic Acid and Resveratrol on Skin Tumor Promotion by 12-O-Tetradecanoylphorbol-13-Acetate. Cancer prevention research.

[24] Kowalczyk MC, Junco JJ, Kowalczyk P, Tolstykh O, Hanausek M, Slaga TJ, Walaszek Z (2013). Effects of combined phytochemicals on skin tumorigenesis in SENCAR mice. International journal of oncology.

[25] Yadav VR, Prasad S, Sung B, Kannappan R, Aggarwal BB (2010). Targeting inflammatory pathways by triterpenoids for prevention and treatment of cancer. Toxins.

[26] Zheng QY, Jin FS, Yao C, Zhang T, Zhang GH, Ai X (2012). Ursolic acid-induced AMP-activated protein kinase (AMPK) activation contributes to growth inhibition and apoptosis in human bladder cancer T24 cells. Biochem Biophys Res Commun.

[27] Checkley LA, Rho O, Angel JM, Cho J, Blando J, Beltran L, Hursting SD, DiGiovanni J (2014). Metformin inhibits skin tumor promotion in overweight and obese mice. Cancer prevention research.

[28] Wu CL, Qiang L, Han W, Ming M, Viollet B, He YY (2013). Role of AMPK in UVB-induced DNA damage repair and growth control. Oncogene.

[29] Huang SW, Wu CY, Wang YT, Kao JK, Lin CC, Chang CC, Mu SW, Chen YY, Chiu HW, Chang CH, Liang SM, Chen YJ, Huang JL, Shieh JJ (2013). p53 modulates the AMPK inhibitor compound C induced apoptosis in human skin cancer cells. Toxicology and applied pharmacology.

[30] Liu J (1995). Pharmacology of oleanolic acid and ursolic acid. Journal of ethnopharmacology.

[31] Liby KT, Sporn MB (2012). Synthetic oleanane triterpenoids: multifunctional drugs with a broad range of applications for prevention and treatment of chronic disease. Pharmacological reviews.

[32] Sheng H, Sun H (2011). Synthesis, biology and clinical significance of pentacyclic triterpenes: a multi-target approach to prevention and treatment of metabolic and vascular diseases. Natural product reports.

[33] Oguro T, Liu J, Klaassen CD, Yoshida T (1998). Inhibitory effect of oleanolic acid on 12-O-tetradecanoylphorbol-13-acetate-induced gene expression in mouse skin. Toxicological sciences: an official journal of the Society of Toxicology.

[34] Allouche Y, Warleta F, Campos M, Sanchez-Quesada C, Uceda M, Beltran G, Gaforio JJ (2011). Antioxidant, antiproliferative, and pro-apoptotic capacities of pentacyclic triterpenes found in the skin of olives on MCF-7 human breast cancer cells and their effects on DNA damage. Journal of agricultural and food chemistry.

[35] Gu G, Barone I, Gelsomino L, Giordano C, Bonofiglio D, Statti G, Menichini F, Catalano S, Ando S (2012). Oldenlandia diffusa extracts exert antiproliferative and apoptotic effects on human breast cancer cells through ERalpha/Sp1-mediated p53 activation. Journal of cellular physiology.

[36] Shan JZ, Xuan YY, Ruan SQ, Sun M (2011). Proliferation-inhibiting and apoptosis-inducing effects of ursolic acid and oleanolic acid on multi-drug resistance cancer cells *in vitro*. Chinese journal of integrative medicine.

[37] Hata K, Hori K, Takahashi S (2002). Differentiation- and apoptosis-inducing activities by pentacyclic triterpenes on a mouse melanoma cell line. Journal of natural products.

[38] Sivakumar G, Vail DR, Nair V, Medina-Bolivar F, Lay JO (2009). Plant-based corosolic acid: future anti-diabetic drug?. Biotechnology journal.

[39] Ahn KS, Hahm MS, Park EJ, Lee HK, Kim IH (1998). Corosolic acid isolated from the fruit of Crataegus pinnatifida var. psilosa is a protein kinase C inhibitor as well as a cytotoxic agent. Planta medica.

[40] Cai X, Zhang H, Tong D, Tan Z, Han D, Ji F, Hu W (2011). Corosolic acid triggers mitochondria and caspase-dependent apoptotic cell death in osteosarcoma MG-63 cells. Phytotherapy research: PTR.

[41] Fujiwara Y, Komohara Y, Ikeda T, Takeya M (2011). Corosolic acid inhibits glioblastoma cell proliferation by suppressing the activation of signal transducer and activator of transcription-3 and nuclear factor-kappa B in tumor cells and tumor-associated macrophages. Cancer science.

[42] Lee MS, Cha EY, Thuong PT, Kim JY, Ahn MS, Sul JY (2010). Down-regulation of human epidermal growth factor receptor 2/neu oncogene by corosolic acid induces cell cycle arrest and apoptosis in NCI-N87 human gastric cancer cells. Biological & pharmaceutical bulletin.

[43] Lee MS, Lee CM, Cha EY, Thuong PT, Bae K, Song IS, Noh SM, Sul JY (2010). Activation of AMP-activated protein kinase on human gastric cancer cells by apoptosis induced by corosolic acid isolated from Weigela subsessilis. Phytotherapy research: PTR.

[44] Xu Y, Ge R, Du J, Xin H, Yi T, Sheng J, Wang Y, Ling C (2009). Corosolic acid induces apoptosis through mitochondrial pathway and caspase activation in human cervix adenocarcinoma HeLa cells. Cancer letters.

[45] Kim JH, Kim YH, Song GY, Kim DE, Jeong YJ, Liu KH, Chung YH, Oh S (2014). Ursolic acid and its natural derivative corosolic acid suppress the proliferation of APC-mutated colon cancer cells through promotion of beta-catenin degradation. Food and chemical toxicology: an international journal published for the British Industrial Biological Research Association.

[46] Lozano-Mena G, Sanchez-Gonzalez M, Juan ME, Planas JM (2014). Maslinic acid, a natural phytoalexin-type triterpene from olives—a promising nutraceutical?. Molecules.

[47] Juan ME, Planas JM, Ruiz-Gutierrez V, Daniel H, Wenzel U (2008). Antiproliferative and apoptosis-inducing effects of maslinic and oleanolic acids, two pentacyclic triterpenes from olives, on HT-29 colon cancer cells. The British journal of nutrition.

[48] Reyes FJ, Centelles JJ, Lupianez JA, Cascante M (2006). (2Alpha,3beta)-2,3-dihydroxyolean-12-en-28-oic acid, a new natural triterpene from Olea europea, induces caspase dependent apoptosis selectively in colon adenocarcinoma cells. FEBS letters.

[49] Wu DM, Zhao D, Li DZ, Xu DY, Chu WF, Wang XF (2011). Maslinic acid induces apoptosis in salivary gland adenoid cystic carcinoma cells by Ca2+-evoked p38 signaling pathway. Naunyn-Schmiedeberg's archives of pharmacology.

[50] Zhang S, Ding D, Zhang X, Shan L, Liu Z (2014). Maslinic acid induced apoptosis in bladder cancer cells through activating p38 MAPK signaling pathway. Molecular and cellular biochemistry.

[51] Villar VH, Vogler O, Barcelo F, Gomez-Florit M, Martinez-Serra J, Obrador-Hevia A, Martin-Broto J, Ruiz-Gutierrez V, Alemany R (2014). Oleanolic and maslinic acid sensitize soft tissue sarcoma cells to doxorubicin by inhibiting the multidrug resistance protein MRP-1, but not P-glycoprotein. The Journal of nutritional biochemistry.

[52] Li C, Yang Z, Zhai C, Qiu W, Li D, Yi Z, Wang L, Tang J, Qian M, Luo J, Liu M (2010). Maslinic acid potentiates the anti-tumor activity of tumor necrosis factor alpha by inhibiting NF-kappaB signaling pathway. Molecular cancer.

[53] Sanchez-Tena S, Reyes-Zurita FJ, Diaz-Moralli S, Vinardell MP, Reed M, Garcia-Garcia F, Dopazo J, Lupianez JA, Gunther U, Cascante M (2013). Maslinic acid-enriched diet decreases intestinal tumorigenesis in Apc(Min/+) mice through transcriptomic and metabolomic reprogramming. PloS one.

[54] Yoo KH, Park JH, Lee DY, Hwang-Bo J, Baek NI, Chung IS (2015). Corosolic Acid Exhibits Anti-angiogenic and Anti-lymphangiogenic Effects on *In Vitro* Endothelial Cells and on an *In Vivo* CT-26 Colon Carcinoma Animal Model. Phytotherapy research: PTR.

[55] Moore T, Beltran L, Carbajal S, Hursting SD, DiGiovanni J (2012). Energy balance modulates mouse skin tumor promotion through altered IGF-1R and EGFR crosstalk. Cancer prevention research.

[56] Rundhaug JE, Fischer SM (2010). Molecular mechanisms of mouse skin tumor promotion. Cancers.

[57] Yoshimura A (2006). Signal transduction of inflammatory cytokines and tumor development. Cancer science.

[58] Mueller MM (2006). Inflammation in epithelial skin tumours: old stories and new ideas. European journal of cancer.

[59] Checkley LA, Rho O, Moore T, Hursting S, DiGiovanni J (2011). Rapamycin is a potent inhibitor of skin tumor promotion by 12-O-tetradecanoylphorbol-13-acetate. Cancer prevention research.

[60] Banno N, Akihisa T, Tokuda H, Yasukawa K, Taguchi Y, Akazawa H, Ukiya M, Kimura Y, Suzuki T, Nishino H (2005). Anti-inflammatory and antitumor-promoting effects of the triterpene acids from the leaves of Eriobotrya japonica. Biological & pharmaceutical bulletin.

[61] Nelson AT, Camelio AM, Claussen KR, Cho J, Tremmel L, DiGiovanni J, Siegel D Synthesis of oxygenated oleanolic and ursolic acid derivatives with anti-inflammatory properties. Bioorganic & Medicinal Chemistry Letters.

[62] Naito M, Naito Y, DiGiovanni J (1987). Comparison of the histological changes in the skin of DBA/2 and C57BL/6 mice following exposure to various promoting agents. Carcinogenesis.

[63] Bozeman R, Abel EL, Macias E, Cheng T, Beltran L, Digiovanni J (2014). A novel mechanism of skin tumor promotion involving interferon-gamma (IFNgamma)/signal transducer and activator of transcription-1 (Stat1) signaling. Molecular carcinogenesis.

[64] Rao D, Macias E, Carbajal S, Kiguchi K, DiGiovanni J (2015). Constitutive Stat3 activation alters behavior of hair follicle stem and progenitor cell populations. Molecular carcinogenesis.

[65] Riggs PK, Angel JM, Abel EL, DiGiovanni J (2005). Differential gene expression in epidermis of mice sensitive and resistant to phorbol ester skin tumor promotion. Molecular carcinogenesis.

