# Supplementary material for: Evaluation of pentacyclic triterpenes found in Perilla frutescens for inhibition of skin tumor promotion by 12-O-tetradecanoylphorbol-13-acetate
Source: Oncotarget. 2015 Oct 15;6(36):39292–306. doi: 10.18632/oncotarget.5751 (PMC4770773; doi:10.18632/oncotarget.5751)
Supplement: Supplementary file 1 [file oncotarget-06-39292-s001.pdf]

SUPPLEMENTARY FIGURE

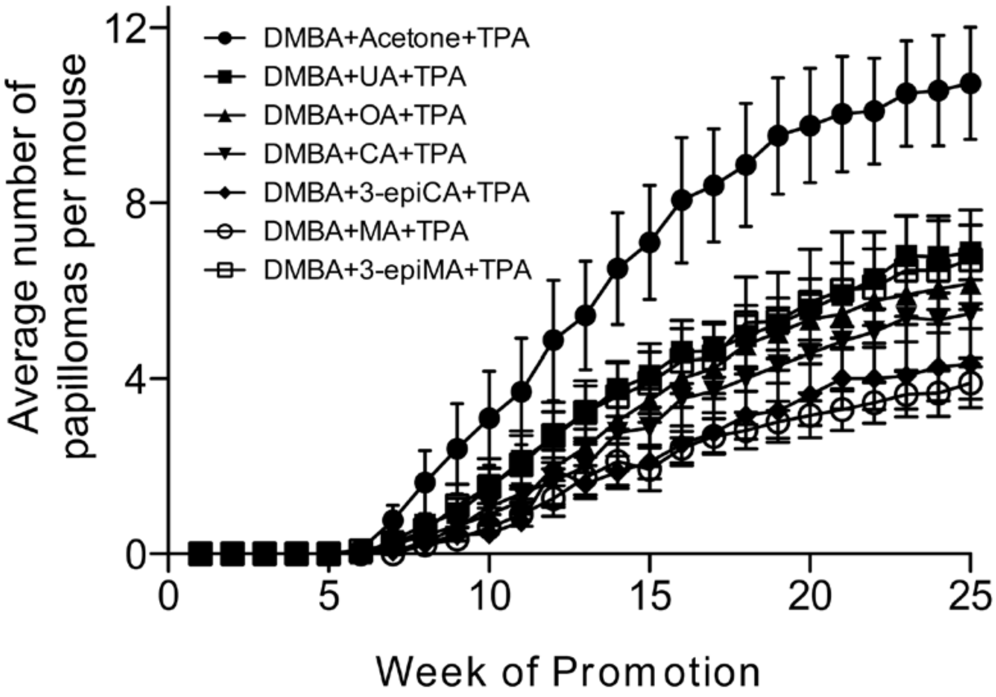

Supplementary Figure S1: Effect of UA and related triterpenes found in *P. frutescens* on tumor multiplicity. Female ICR mice 7 weeks old (*n* = 30/group) were initiated with 25 nmol DMBA. Two weeks after initiation with DMBA, mice were pretreated with either acetone vehicle (0.2 ml), UA (2 µmol), OA (2 µmol), CA (2 µmol), 3-epiCA (2 µmol), MA (2 µmol) and 3-epiMA (2 µmol) 30 min prior to each 6.8 nmol TPA treatment. All treatments were given twice-weekly. Tumor multiplicity was measured once a week for 25 weeks. Each data point on the graph shows the average number of papillomas per mouse ± standard error of the mean (SEM).
